# Towards a Better Understanding of Microbial Community Dynamics through High-Throughput Cultivation and Data Integration

**DOI:** 10.1128/mSystems.00101-19

**Published:** 2019-05-28

**Authors:** Karoline Faust

**Affiliations:** a KU Leuven Department of Microbiology, Immunology and Transplantation, Laboratory of Molecular Bacteriology, Rega Institute, Leuven, Belgium

**Keywords:** community models, data integration, high-throughput cultivation, microbial community dynamics, microbial systems biology

## Abstract

The investigation of microbial community dynamics is hampered by low resolution, a lack of control, and a small number of replicates. These deficiencies can be tackled with defined communities grown under well-controlled conditions in high-throughput automated cultivation devices. Besides delivering high-quality microbial community data, automated cultivation will also ease measurement of the basic parameters needed to parameterize mathematical models of microbial communities. Better experimental data will allow revisiting classical ecological questions, such as the impact of community structure on dynamics. In addition, such data will allow validation and comparison of community models and benchmarking of microbial data analysis software. In summary, high-throughput automated cultivation will lead to a deeper understanding of microbial community dynamics through better models and software.

## Abstract (precis)

The investigation of microbial community dynamics is hampered by low resolution, a lack of control, and a small number of replicates. These deficiencies can be tackled with defined communities grown under well-controlled conditions in high-throughput automated cultivation devices.

## PERSPECTIVE

Microbial ecosystems are complex, often consisting of hundreds of member species that interact with each other and with the environment in intricate ways, making their investigation a challenging endeavor. While many microbial communities have been sequenced in the last 2 decades, few studies have gone beyond microbial composition and associations. Thus, we still do not understand well the rules governing microbial community dynamics. Yet such an understanding is crucial when manipulating microbial communities, for instance, to optimize consortia for certain tasks such as toxin degradation (1) or to shift gut microbiota toward more healthy states (2).

## TOP-DOWN VERSUS BOTTOM-UP EXPLORATION OF MICROBIAL ECOSYSTEMS

There are two main avenues to explore microbial ecosystems: the top-down approach investigates a microbial ecosystem in its entirety. It is, for instance, represented by 16S studies of the human gut microbiota (3). Due to the many unknown factors and lack of control, top-down studies usually remain at the descriptive level, delivering associations rather than causal relationships. In contrast, the bottom-up approach simplifies the system to study selected aspects in more depth, with a higher level of control than can be achieved *in situ*. For instance, a bottom-up approach has led to the formulation of the competitive exclusion principle (4). Since there is a trade-off between studying a full versus a simplified ecosystem and gaining descriptive versus causal insight, both approaches are valuable. However, given the overwhelming number of sequencing studies versus the few *in vitro* studies, they are currently not well balanced. Thanks to recent developments in high-throughput cultivation such as minibioreactor arrays (5) and microfluidic flow cells (6), bottom-up studies are currently increasing in number. These next-generation cultivation devices allow researchers to carry out many controlled experiments in parallel. Combined with recent advances in the quantification of microbial abundances and metabolites, this means that we can study microbial ecosystems at an unprecedented depth, giving us the opportunity to address a number of old ecological questions in new ways.

## QUESTIONS ABOUT MICROBIAL COMMUNITIES AND THEIR REPRESENTATIONS

A number of classical questions pertain to the link between community structure and dynamics. How do species number, proportions, and interactions shape the dynamics? Do some community structures resist perturbations better or recover more quickly than others and, if so, why? Other questions address mechanisms behind phenomena: what drives alternative community states, and how can we distinguish between different mechanisms explaining them? Are there species that are more important than others for the maintenance of a particular community composition and, if so, which properties make them keystones (7)?

These questions are rarely addressed experimentally, but are often tackled *in silico* using mathematical representations of ecosystems (e.g., see reference 8). Microbial ecosystems can be modeled at different levels of resolution (e.g., populations, individuals, and biochemical reactions within individuals) and by making different assumptions (e.g., on the role of interactions, immigration, or the environment). Thus, there are also questions related to the representation of ecosystems. Are the model assumptions justified? How accurate are model predictions? Do simple models predict ecosystem dynamics sufficiently well for the purpose at hand, or are more complex models with more parameters required? Do properties of ecosystem representations (such as hub nodes in networks) predict properties of ecosystems (such as keystone species)? How and with what accuracy can we derive model parameters from observational data?

**FIG 1:**
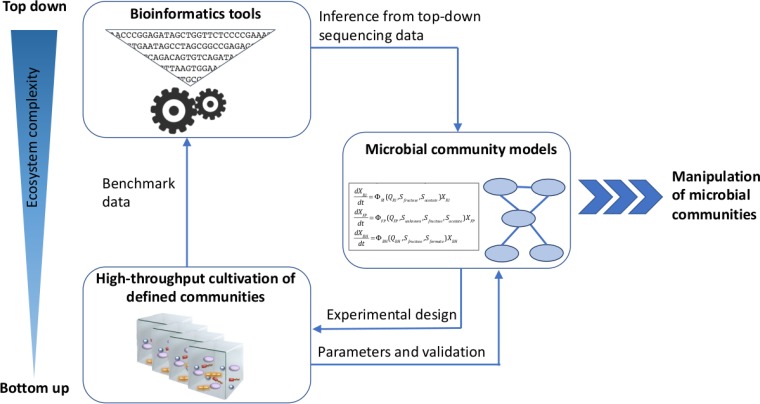
Overview of the interplay between selected top-down and bottom-up approaches for the exploration of microbial ecosystems. Bioinformatics tools dedicated to the analysis of community sequencing data represent a top-down approach, whereas defined communities grown under controlled conditions *in vitro* are an example of a bottom-up approach. Ecological hypotheses in the form of community models can be derived from both approaches. However, the bottom-up approach provides in addition data to benchmark bioinformatics tools and parameters needed for many community models. The ecological insight encoded in validated community models eases the manipulation of microbial communities.

I plan to dedicate my research to these questions, which I intend to address with both bottom-up and top-down approaches. To implement bottom-up approaches, I plan to establish defined microbial communities under well-controlled conditions and monitor them in many replicates (started on a small scale [9]). Following the paradigm of systems biology, these *in vitro* data will be used to improve and test community models. In addition, I aim to pursue top-down research by developing bioinformatics tools that extract ecological hypotheses from microbial community data. Both research lines cross-fertilize: *in vitro* experiments will provide benchmarks for bioinformatics tools and community models, which in turn deliver hypotheses that guide *in vitro* experiments (Fig. 1).

## DEFINED COMMUNITIES IN MANY REPLICATES YIELD NEW ECOLOGICAL INSIGHTS

The advantage of growing defined communities of known composition in a controlled environment is that mathematical models can account for all factors that may impact the dynamics, which is not possible in other settings. Depending on the degree of stochastic variability and the presence of chaotic behavior, microbial community models can make generic or precise predictions (e.g., on abundance distributions, the composition of stable states, and responses to perturbations). In a highly controlled system, a discrepancy between these predictions and observation is clearly attributable to a lack of understanding and not to hidden environmental factors or unobserved species. Thus, defined communities allow the discovery of phenomena that are hard to discern from top-down studies.

While defined communities are becoming more popular (e.g., see reference 10), the number of replicates is usually still low. A large number of replicates is needed—not only to reach statistical significance. The breakthrough paper by Hekstra and Leibler, where the authors investigate the reproducibility of ecosystem dynamics, demonstrates that a large number of replicates is a source of insight in its own right (11). If we want to explore phenomena such as multistability or stochasticity, we need to be able to assess biological variation and to disentangle it from meaningless technical variation. In my research, I aim to achieve this goal by combining automated high-throughput cultivation with improved cell counting techniques.

## HIGH-THROUGHPUT CULTIVATION EASES MODEL PARAMETERIZATION

Another advantage of automated high-throughput cultivation is the efficient and accurate measurement of parameters such as pH preferences, growth rates, interaction strengths, nutrient uptake and production rates, and so on. Although these parameters are a prerequisite for modeling, comprehensive measurements are still lacking. Extending our knowledge of basic microbial physiology will also help to improve metabolic reconstructions, which are needed for metabolic models. Since metabolic models take each biochemical reaction in each community member into account, they span several levels of organization, ranging from genes to ecosystems. For this reason, metabolic models are one of the most promising tools to engineer microbial consortia (e.g., see reference 1). However, metabolic models are rarely systematically validated. It is therefore still an open question whether they fulfill their promise at the community level. Assessment of the performance of metabolic models will be an important part of my bottom-up research agenda.

## DATA INTEGRATION IS A CHALLENGE FOR SEQUENCING DATA ANALYSIS TOOLS

Another strength of metabolic models is their potential to integrate different data sources (e.g., see reference 12), an ability that is still missing in many of the current bioinformatics tools dedicated to the analysis of microbial sequencing data. The endpoint of the analysis of such data is often a multivariate visualization in the form of an ordination plot or an association network. The integration of metabolic, physiological, and environmental data obtained from various sources is crucial to interpret these results. In many cases, the bottleneck is not the availability of such data, but the effort required to fully exploit them. My top-down research plan therefore includes the development of tools that make better use of available data to extract ecological hypotheses from sequencing studies.

## OUTLOOK

In the next 5 years, I expect that we will be able to monitor simplified microbial ecosystems routinely with unprecedented accuracy, resolution, and number of replicates. This will mean that we can systematically query ecosystem behavior in a way previously feasible only through simulations *in silico*. In my opinion, this will allow optimizing low-complexity communities for a broad range of tasks, including bioremediation, biofuel, and food production.

The next step will then be to scale mathematical models so they can be applied to complex communities in top-down studies. While the gap between *in vitro* and *in situ* is routinely bridged experimentally with mesocosms and, for host-associated microbiota, with gnotobiotic animals, many of the community models cannot tackle more than a few species. Guild-level prediction at the mesocosm scale can be accurate (e.g., see reference 13), but is not sufficient for all applications. Thus, much work is still needed to develop, parameterize, and validate species-level models that can be applied to complex communities in heterogeneous environments.

Improved data collection has often sparked new scientific insights. The investigation of microbial ecosystems in a well-controlled high-throughput manner has a good chance to become another case in point.
